# Auxin-mediated biosynthesis of silver nanoparticles: comprehensive characterisation and antibacterial activity analysis

**DOI:** 10.3389/fcimb.2025.1678489

**Published:** 2025-11-17

**Authors:** Sahil, Anis Ahmad Chaudhary, Sonia Sorout, Kushi Yadav, Mohamed A. M. Ali, Fehmi Boufahja, Vikram Kumar, SL Kothari, Devendra Jain, Kumar Sambhav Verma

**Affiliations:** 1 Amity Institute of Biotechnology, Amity University Rajasthan, Jaipur, Rajasthan, India; 2 Department of Biology, College of Science, Imam Mohammad Ibn Saud Islamic University (IMSIU), Riyadh, Saudi Arabia; 3 Amity Institute of Biotechnology, Amity University Uttar Pradesh, Noida, Uttar Pradesh, India; 4 Amity Institute of Pharmacy, Amity University Rajasthan, Jaipur, Rajasthan, India; 5 Department of Molecular Biology and Biotechnology, Maharana Pratap University of Agriculture & Technology, Udaipur, Rajasthan, India

**Keywords:** nanoparticles, antimicrobial activity, antibiotic, indole-3-acetic acid, indole-3-butyric acid, green nanotechnology, auxin-mediated synthesis

## Abstract

**Background:**

Silver nanoparticles (AgNPs) are well-known for their potent antibacterial properties. However, the rise of antibiotic-resistant bacteria highlights the need for alternative antimicrobial strategies. Green synthesis using biological molecules offers an eco-friendly route to nanoparticle production.

**Objective:**

This study aims to synthesise AgNPs using plant growth hormones (Auxins), specifically indole-3-acetic acid (IAA) and indole-3-butyric acid (IBA), and to evaluate their antibacterial activity against pathogenic bacteria.

**Methods:**

AgNPs were synthesised using IAA and IBA as reducing and stabilising agents. The synthesised nanoparticles were characterised by UV-Visible spectroscopy, Fourier Transform Infrared Spectroscopy (FTIR), Particle Size Analysis, Dynamic Light Scattering (DLS) for hydrodynamic diameter, Zeta potential for surface charge, and Field Emission Scanning Electron Microscopy (FE-SEM) for morphological analysis. Antibacterial assays were performed against *Staphylococcus aureus* and *Escherichia coli*.

**Results:**

The IAA and IBA-mediated AgNPs showed controlled size distribution, stability, and uniform morphology. Enhanced antibacterial activity was observed, particularly against *S. aureus*, compared to *E. coli*.

**Conclusion:**

IAA and IBA-mediated synthesis provide a green, sustainable method for producing AgNPs with significant antibacterial potential. These Auxin-based AgNPs represent promising candidates for combating antibiotic-resistant bacterial strains.

## 1 Introduction

Nanobiotechnology merges nanoscience with biotechnology, focusing on biomedical applications. Nanoparticles, with unique physical and chemical properties, are crucial in drug delivery, diagnostics, and therapeutic devices (Roco, 2003). They are used in medical imaging, tissue engineering, and targeted treatments, offering enhanced precision and biocompatibility (Mansour et al., 2023). Whether artificially produced, naturally occurring, or accidental, nanoparticles are driving advancements in medical technologies, with growing applications in healthcare and pharmaceuticals (Haleem et al., 2023). Many socioeconomic advancements have resulted from increased interest in engineered nanoparticles such as silver, copper, and gold in various scientific fields, including medicinal, materials, and agricultural technology (Zakaria et al., 2015; Chernousova and Epple, 2013). Similarly, silver has been used as an antibacterial agent in multiple ways, either by itself or in conjunction with other technologies (Silva et al., 2017). Silver’s antibacterial property was discovered, prompting researchers to investigate AgNPs’ antibacterial potential in nanotechnology (Rizzello and Pompa, 2013). AgNPs fall within the category of nanomaterials, having a size range between 1 to 100 nanometers. These materials outperform silver in terms of surface area-to-volume ratio and overall capacity. This material is appropriate for targeted drug delivery, diagnostics, detection, and imaging because it has special electrical, optical, and catalytic properties at the nanoscale (Yaqoob et al., 2020). Due to the excellent antibacterial properties of AgNPs, they have been given significant attention by both the industry and academia.

AgNPs possess potent antimicrobial properties toward a broad spectrum of harmful and infectious pathogens, including multidrug-resistant bacteria (Siddiqi et al., 2018; Marambio-Jones and Hoek, 2010). As AgNPs exhibit greater antibacterial action at the nanoscale, they have applications in medical and healthcare fields such as dressings, surgical instruments and dental products (Kulkarni, 2014; Ge et al., 2014). AgNPs can target multiple microorganisms at once; they have the potential to eradicate a broad spectrum of bacteria, which makes them promising as antibiotics (Cheng et al., 2016; Betts et al., 2018). With antibiotic resistance rising—a global health threat responsible for an estimated 1.27 million deaths worldwide in 2019 alone developing new antimicrobial strategies is urgent (Salam et al., 2023). Creating new antibiotics takes a lot of time and resources, and years of research are needed to guarantee their safety and efficacy. Meanwhile, diseases caused by microbes resistant to several drugs continue to be fatal worldwide (Natan and Banin, 2017; Lee et al., 2019). Researchers have investigated employing AgNPs and other nanomaterials to attack hazardous microorganisms in the post-antibiotic era without creating resistance (Betts et al., 2018). AgNPs offer a promising solution for preventing infections, decontaminating medical supplies, and fighting diseases despite the global problem of antibiotic-resistant microorganisms (Štefančič et al., 2005; Pop et al., 2011). Recent research has concentrated on creating novel bactericidal chemicals for decontamination or infection therapies to combat multidrug-resistant pathogens (Natan and Banin, 2017).

In the present study, we synthesised silver nanoparticles by using biomolecules, Auxins, including indole-3-acetic acid (IAA) and indole-3-butyric acid (IBA) as reducing cum capping agents. This simple process needs a few simple steps and equipment and is quick and affordable. Silver nanoparticles with different sizes and distributions can be synthesised. The ideal method for creating stable AgNPs with the required optical properties was tried using a variety of synthesis variables. UV-Vis Spectroscopy has studied the synthesised AgNPs, Fourier transform infrared spectroscopy (FTIR), Field Emission Electron Microscopy (FE-SEM) and additionally, the antibacterial effects of synthesised nanoparticles (AgNPs) on *Staphylococcus aureus* (*S. aureus*) and *Escherichia coli* (*E. coli*) were analysed. The results demonstrated the strong antibacterial effect of AgNPs on these two bacteria.

## 2 Materials and methodology

### 2.1 Material required

Using IAA and IBA as reducing agents, precursor silver (AgNO3) was reduced to Ag0, while KOH served as an alkaline medium. All the chemicals were procured from Sigma-Aldrich Co., Ltd. in the United States, and the solvent was Milli-Q water with a conductivity of 18.2 MΩ.

### 2.2 Preparation of silver nanoparticles

A solution containing 50 mL of AgNPs was made. It comprised 5 mL of 10 mM IAA or IBA, 1 mL of 1 mM AgNO3, 860 μl of 10 mM potassium hydroxide (KOH), and Milli-Q water comprised the remaining volume. Using a magnetic stirrer set at 60°C, 860 μl of a 10 mM potassium hydroxide solution (KOH) was added drop by drop to a flask containing 43 ml of deionised water for the synthesis process. IAA and IBA solutions were added separately and served as the reaction’s starting point to stop aggregation. AgNO3 solution was then added gradually, drop by drop. The colour shift from translucent to yellow, which was thought to be the reaction’s end point, indicates the formation of colloidal nanoparticles stabilised by IAA and IBA. Additionally, the mixture was incubated for three hours at 60°C on a magnetic stirrer to ensure the nanoparticles were dispersed correctly.

### 2.3 Characterisation of IAA- and IBA-stabilised AgNPs

Understanding the physicochemical characteristics of nanoparticles, including their size, shape, size distribution, dispersion, surface charge, and interaction mechanisms, requires thorough characterisation and analysis.

A UV-Vis Spectrometer (Thermo Scientific Multiskan GO) was used to record the UV-Vis spectra of AgNPs stabilised with IAA and IBA. In contrast, pure IAA and IBA solutions were used as controls. AgNPs practical synthesis was validated by a pronounced surface plasmon resonance (SPR) band feature. The solutions were centrifuged at 10, 976 × g for 10 minutes at 15 °C to remove surplus stabilising agents to ensure that only the stabilised nanoparticles contributed to the characterisation results. The size distribution and optical characteristics of the particles were revealed by the SPR study, which is essential for assessing their colloidal behaviour. The hydrodynamic diameter of the AgNPs was ascertained using Dynamic Light Scattering (Litesizer 500 Anton Par, Austria), using a backscatter detector with an angle of 15˚, 90˚, and 175˚ at 25 °C. The hydrodynamic size differences between IAA-and IBA-stabilised AgNPs were demonstrated by the DLS data, with IAA formulations showing somewhat broader size distributions than the more homogeneous sizes seen with IBA. Zeta potential tests, which were also carried out with the DLS system, revealed information about the stability and surface charge of the nanoparticles. Zeta potential values above ±25 mV were shown by both formulations, suggesting superior colloidal stability and long-term aggregation resistance.

### 2.4 Antibacterial testing of AgNPs

#### 2.4.1 Morphological and structural characterization of IAA- and IBA-Stabilized silver nanoparticles via FE-SEM and FTIR spectroscopy

The AgNPs shape and structural characteristics were examined using Field Emission Scanning Electron Microscopy (FE-SEM). To avoid sample overloading, the IAA and IBA-stabilised AgNP solutions were diluted with Milli-Q water at a 1:20 ratio. On a sanitised, glass-covered slide, a 2 μL drop of the diluted solution was placed and left to air dry at room temperature. A thin layer of silver was applied to the dried samples using a sputter coater to improve conductivity. A FE-SEM (TESCAN MIRA, Brno, Czech Republic) with a secondary electron detector running at a 25 keV accelerating voltage was used to analyse the materials.

Fourier-transform infrared (FT-IR) spectroscopy (ABB MB 3000) was used to examine the molecular interactions between the AgNPs and the stabilising agents (IAA and IBA). For FT-IR measurement, the stabilised AgNPs were centrifuged for 10 minutes at 14, 000 RPM to extract the nanoparticles, which were then ground into a fine powder using potassium bromide (KBr). Pure IAA and IBA were used as control samples, and the spectra were captured in the 400–4000 cm⁻¹ range. IAA and IBA play crucial roles in the synthesis and stabilisation of nanoparticles, as demonstrated by the FTIR analysis’s identification of functional groups, including hydroxyl, carboxyl, and indole groups engaged in the reduction and stabilisation processes.

#### 2.4.2 Minimum inhibition concentration assay

The Well diffusion method determined the antibacterial activity against *S. aureus* and *E. coli*. Before microbiological studies, all culture medium, glassware, containers, and microtips were autoclaved at 121^0^C for 15 minutes. *S. aureus* and *E. coli* were cultured in LB broth at 37^0^C until the optical density at 600 nm was observed to be 0.6. Wells were created by puncturing solidified agar plates. 100 μL of culture was spread on the plates using an L-shaped spreader. Samples included AgNP (5ppm) and Ampicillin (1 mg/ml) as positive control, IAA (5ppm), IBA (5ppm) and water as negative control, and IAA-AgNPs (5ppm), IBA-AgNPs (5ppm) as test samples. 20 μL of control and 20, 50, and 100 μL of test sample were placed in their respective wells to observe concentration-dependent antibacterial action. Plates were incubated for 16 hrs at 37^0^C. The reaction was performed in triplicate to maintain the reproducibility and reliability of the results.

#### 2.4.3 Time-dependent antibacterial assay

The concentration range was selected from the preliminary MIC test. 200 μL of 0.5 OD *E. coli* were added to each well of the 96-well plates. 20 μL samples were added to the well containing *E. coli*. Autoclaved Mili-Q was added to *E. coli* as a positive control. Ampicillin was added as a negative control. AgNP served as a reference while Ag-IAA and Ag-IBA were the test samples. A time-dependent assay with a total time of 8 hrs was performed. Hourly reading was taken at 600 nm through Thermo Scientific Multiskan GO. A reading of 0 hrs was subtracted from all the subsequent readings associated with it to obtain the growth of bacterial cells. A similar experiment was repeated with *S. aureus* to confirm growth inhibition in gram-positive bacteria.

#### 2.4.4 Statistical analysis

All the experiments were carried out using two-way ANOVA was performed to analyse the effects of IAA and IBA stabilised AgNPs to determine the significance of antimicrobial activity, while other methods assess parameters like MIC (Minimum Inhibitory Concentration) and MBC (Minimum Bactericidal Concentration). Statistical analysis helps researchers understand the relationship between nanoparticle properties (size, shape, concentration, etc.) and their antimicrobial effects, leading to more efficient and targeted applications.

The analysis was performed using Graphpad Prism included two independent variables: treatment time (a row factor) and experimental condition (a column factor), and their interaction. A percentage contribution of each factor as a source of variation was calculated, and a significance level of p ≤ 0.0001 was established.

## 3 Results and discussion

**Figure 1:**
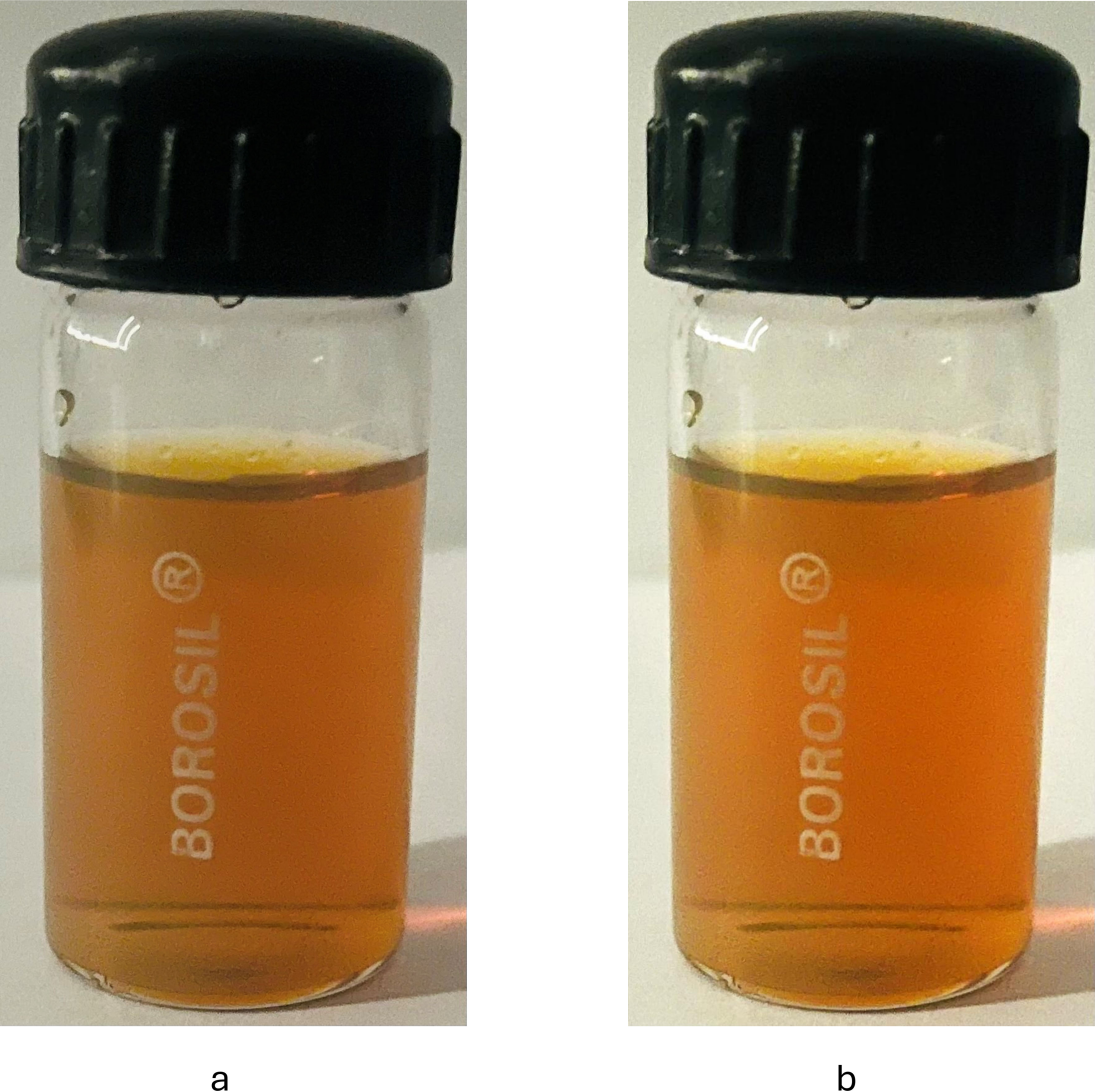
Image of silver nanoparticles **(a)** IAA-AgNPs **(b)** IBA-AgNPs.

As the reaction proceeded, the colour changed to a deeper yellowish brown, indicating the reduction of silver ions and the formation of nanoparticles, thus, the conversion of Ag+ to Ag0 (**Figure 1**). This colour change is characteristic of the surface plasmon resonance (SPR) effect associated with silver nanoparticles. The impact of this synthesis of AgNPS was monitored in a Vis spectrophotometer.

**Figure 2:**
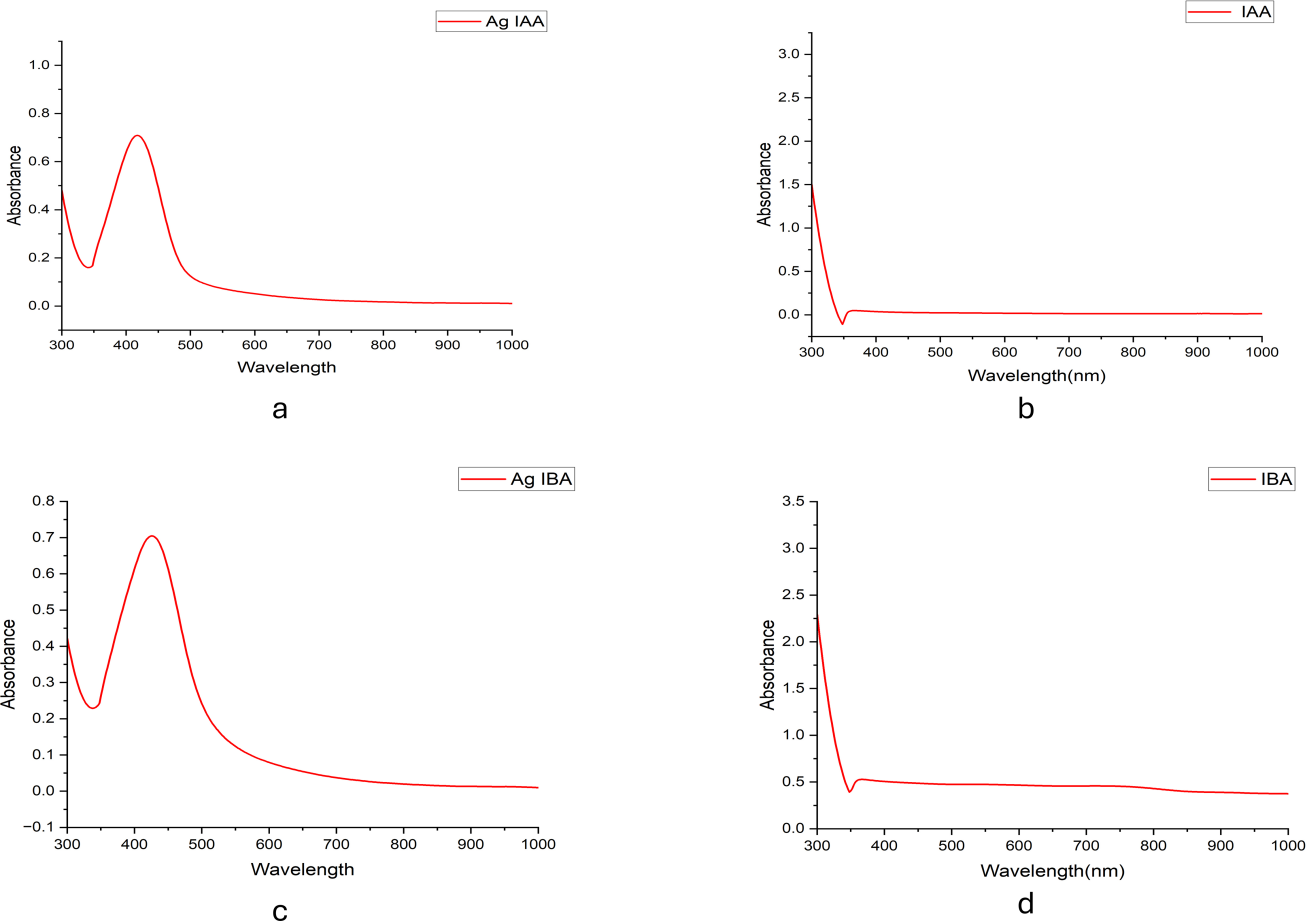
UV -Visible spectra of **(a)** Ag -IAA, **(b)** IAA, **(c)** Ag -IBA, and **(d)** IBA. Distinct SPR peaks around 400 -450 nm in Ag -IAA and Ag -IBA confirm silver nanoparticle formation.

UV-visible spectroscopy further confirmed this phenomenon, which showed clear absorption peaks for AgIAA and AgIBA at about 420 nm (**Figure 2**). The effective reduction of Ag+ to AgNPs by IAA and IBA was confirmed by this SPR band, which is indicative of spherical or nearly spherical nanoparticles. A range of particle sizes influenced by the concentration of the respective plant hormones was suggested by the absorption maxima (λ_max_) seen beyond 300 nm in both IAA- and IBA-stabilised colloids. Higher concentrations facilitated the creation of larger nanoparticles, whereas lower concentrations encouraged the formation of smaller ones, with IAA and IBA acting as stabilising and reducing agents.

**Figure 3:**
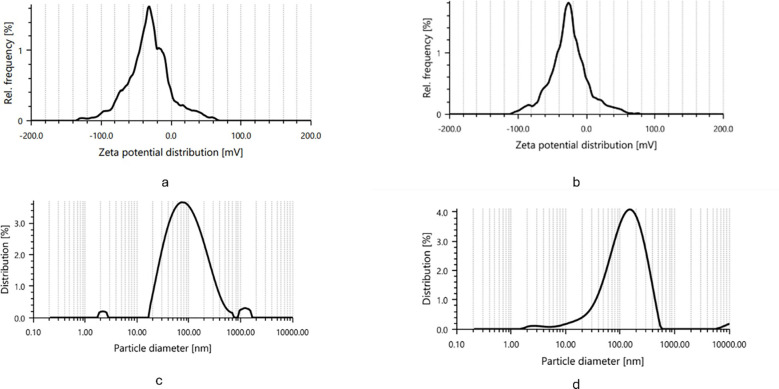
Zeta potential and particle size distribution of synthesized silver nanoparticles. **(a, c)** IAA -AgNPs and **(b, d)** IBA -AgNPs showing stable zeta potential and uniform particle size distribution in the nanometer range.

Dynamic light scattering (DLS) analysis provided insights into the size distribution of nanoparticles. As revealed in **Figure 3**, IBA-AgNPs ranged between 10 and 100 nm, with an average diameter of 364 nm. IAA-AgNPs had a size range of 10–1000 nm, with an average diameter of 188.03 nm. The DLS histograms emphasised IAA-AgNPs polydispersity from aggregation, while IBA-AgNPs had a more uniform size, indicating more stability.

These results were corroborated by zeta potential measurements, which showed that the mean zeta potential for IAA-AgNPs was -28.2 mV and for IBA-AgNPs, it was -28.9 mV. Strong electrostatic repulsion was demonstrated by these values, guaranteeing colloidal stability. The structural characteristics of IBA most likely contributed to the increased stability of IBA-AgNPs (**Figure 3**).

**Figure 4:**
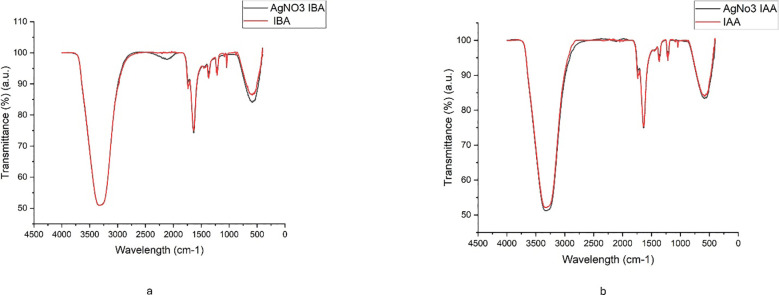
FTIR spectra of **(a)** IBA and AgNO3 -IBA, and **(b)** IAA and AgNO3 -IAA, showing characteristic functional groups involved in silver nanoparticle synthesis and stabilization.

**Figures 4a, b** show Fourier-transform infrared (FTIR) spectroscopy, confirming the conjugation of the plant hormones to the nanoparticles. The IBA-AgNPs spectrum shows characteristic peaks near 1710 cm⁻¹ and 1600 cm⁻¹, corresponding to the carbonyl (C=O) stretching vibrations of IBA. Similarly, the IAA-AgNPs spectrum displays peaks around 1700 cm⁻¹ and 1600 cm⁻¹ attributed to the carbonyl groups of IAA. Both spectra feature broad absorption bands between 3200 and 3500 cm⁻¹, which are assigned to the –OH stretching vibrations from residual water or the hydroxyl groups of the plant hormones. These distinctive spectral features strongly support the effective binding and stabilization of AgNPs by IBA and IAA molecules.

**Figure 5:**
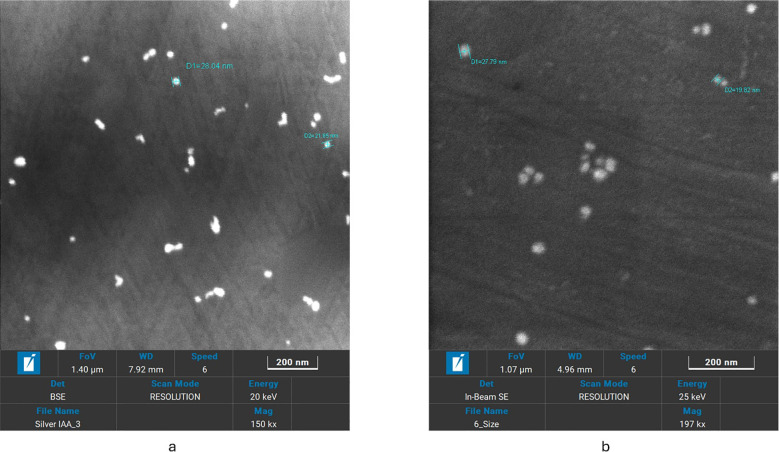
SEM images of **(a)** IAA -AgNPs and **(b)** IBA -AgNPs showing uniformly dispersed nanoparticles.

In **Figures 5a, b**, uniformly distributed spherical nanoparticles were visible in the Fe-SEM pictures. IBA-AgNPs showed a smaller size range (6–70 nm), but IAA-AgNPs showed a wider size range (20–150 nm). This discrepancy explains how IAA and IBA interact molecularly with the nanoparticle’s surface. While IBA-AgNPs smaller size range showed steric and electronic impacts of IBA molecular structure, IAA-AgNPs wider size distribution suggested varying nucleation and growth settings. It was also observed that dilution plays a unique role in producing high-resolution photographs.

### 3.1 Antibacterial testing of silver nanoparticles

**Figure 6:**
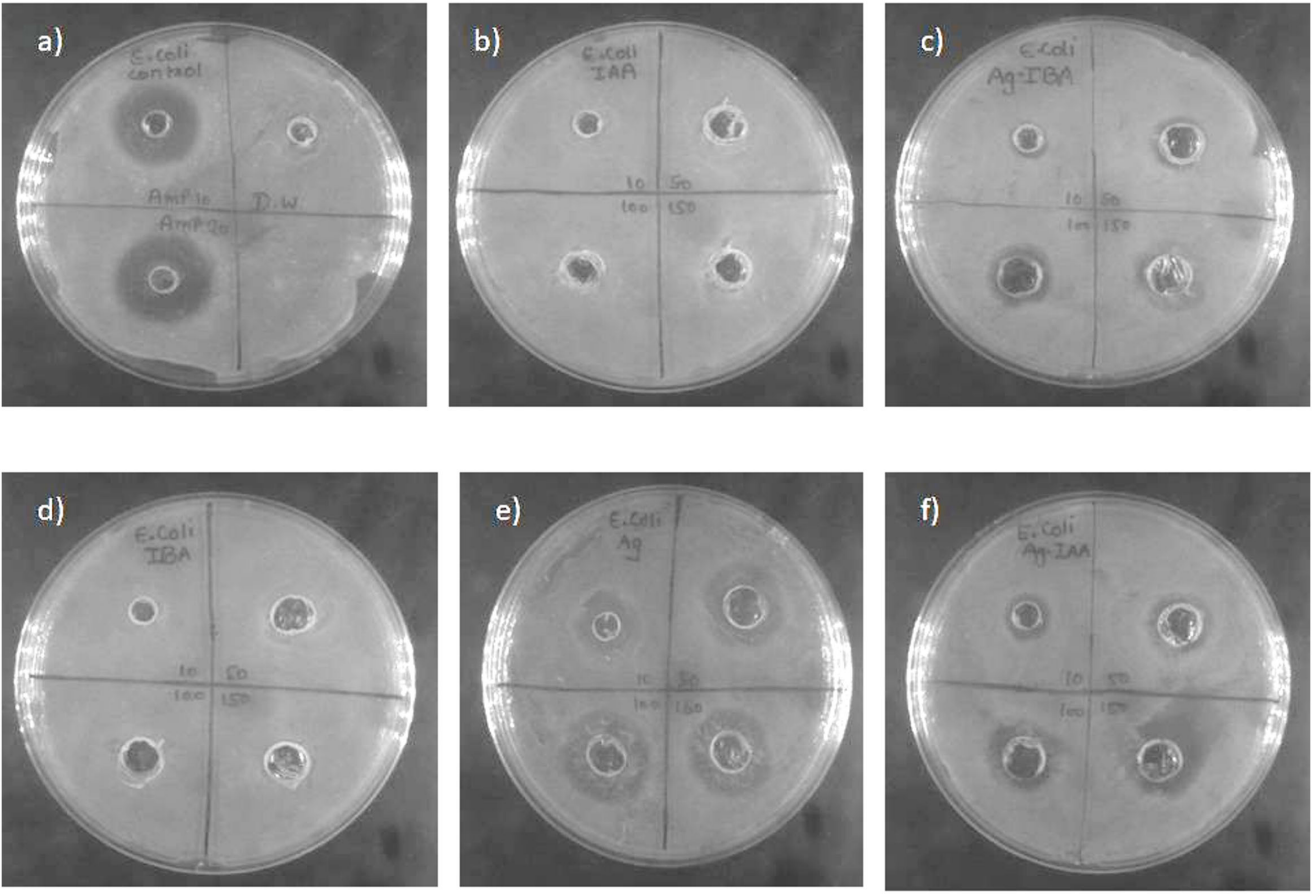
Antibacterial activity of silver nanoparticle of *E. coli***(a)** Control -Ampicillin and Distilled water, **(b)** IAA, **(c)** IBA-AgNPs, **(d)** IBA, **(e)** Ag, **(f)** IAA-AgNPs.

**Figure 7:**
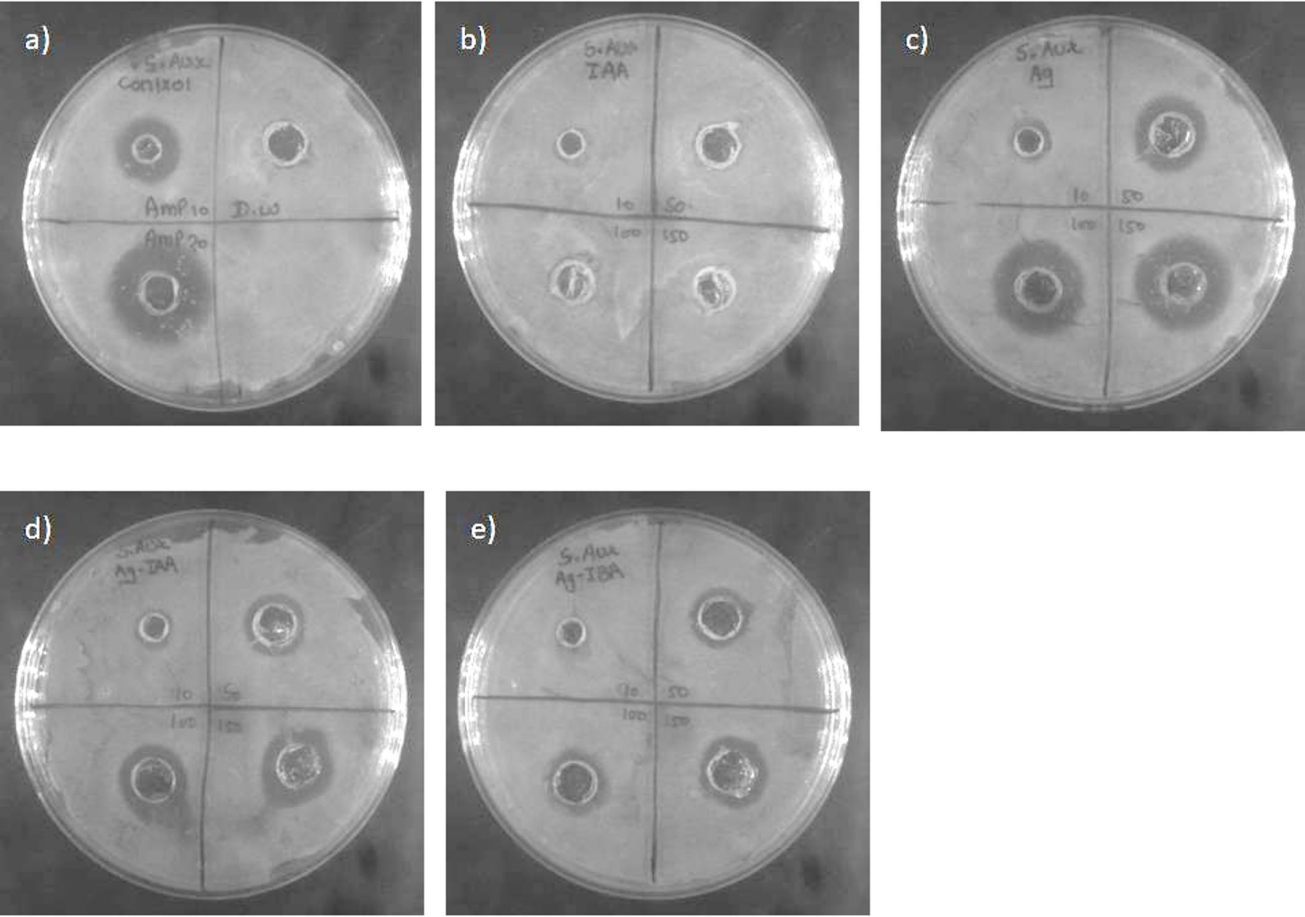
Antibacterial activity against *S. aureus*: **(a)** control, **(b)** IAA, **(c)** Ag, **(d)** Ag -IAA, and **(e)** Ag -IBA showing enhanced inhibition with Ag -IAA and Ag -IBA.

**Figures 6**, **7** AgNPs synthesised with IAA and IBA exhibit antibacterial properties. At 10 ppm IAA and IBA AgNPs did not show an inhibition zone in the case of *E. coli* and *S. Aureus*. However, 50, 100 and 150 ppm IAA and IBA AgNPs showed clear inhibition zones in *E. coli* and *S. aureus* in a concentration-dependent manner. Neither IAA nor IBA showed any inhibition zone in *E. Coli* and *S. aureus*, implying that growth hormone does not possess antibacterial properties.

**Figure 8:**
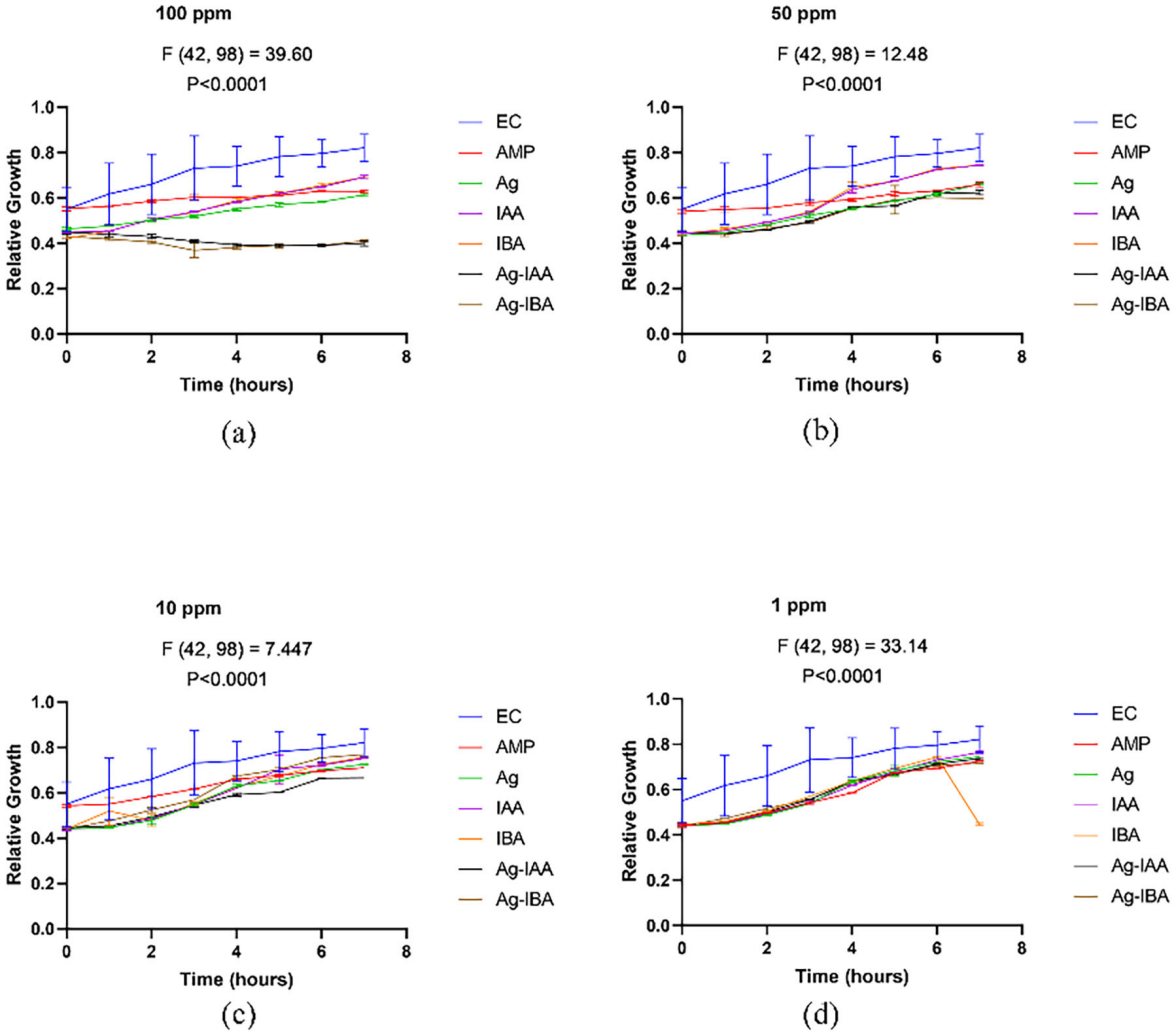
Time and concentration depend on studies of antimicrobial activity of IAA/IBA-AgNPs on *E*. *coli***(a)**100 ppm, **(b)** 50 ppm, **(c)** 10 ppm, **(d)** 1 ppm.

**Figure 9:**
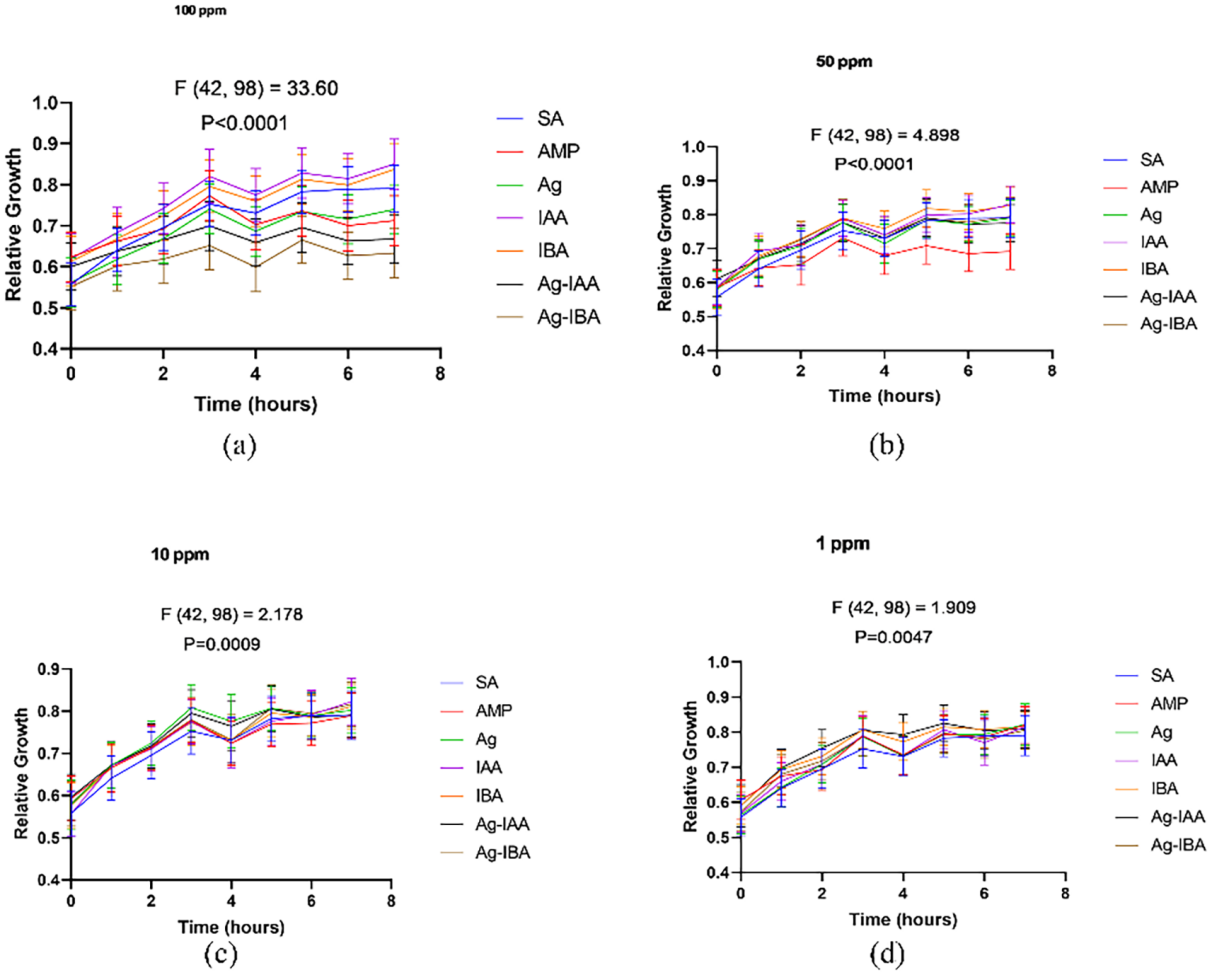
Time and concentration dependent studies of antimicrobial activity of IAA/IBA-AgNPs on S. *aureus***(a)**100 ppm, **(b)** 50 ppm, **(c)** 10 ppm, **(d)** 1 ppm.

With the disc diffusion method, a time concentration dependent assay was conducted to demonstrate the antimicrobial activity of IAA and IBA AgNPs. In *E. coli* (**Figure 8**), both 100 ppm and 50 ppm of IAA/IBA AgNP exposure resulted in a significant decrease in the cell population with the OD values decreasing over time and nearly 60–70% compared to the control. At the 10 ppm and 1 ppm concentrations there was no effect on the growth of *E. coli* with the growth curves overlapped with untreated control. For *S. aureus* (**Figure 9**), both 100 ppm and 50 ppm of AgNPs permitted some limited growth during the first hours whereas the subsequent period decreased in cell population with a relative ~50–65% reduction of microbial cells from the control. As for *E. coli*, the 10 ppm and 1 ppm concentrations did not have an effect on the growth of *S. aureus*.

## 4 Discussion

AgNPs are synthesised by utilising IAA and IBA as reducing agents to transform silver ions into nanoparticles, because these AgNPs break down cell walls and produce reactive oxygen species. They have significant antibacterial effects on various pathogens, including Gram-positive bacteria like *S. aureus* and Gram-negative bacteria like *E. coli.* (Ahmad et al., 2003). Their potential for combination therapy is clear and presents a chance to improve on existing treatments. Still, an extensive approach is required to guarantee their safety and effectiveness, considering their cytotoxicity and hemocompatibility. To further enhance their effectiveness and to overcome resistance, combined therapy with traditional antibiotics should be explored. However, to guarantee safety and biocompatibility, it is essential to evaluate these nanoparticles’ genotoxicity, cytotoxicity, and hemocompatibility (Ackerley et al., 2004).

AgNPs made by IAA and IBA exhibit strong inhibitory effects against *S. aureus* and *E. coli* regarding their antibacterial activity. Our results show that IAA- and IBA-stabilized AgNPs exhibit enhanced antibacterial activity, particularly against *E. coli*. The previous studies suggest that AgNPs may exert their antibacterial effects through mechanisms like ROS generation, membrane damage, and protein denaturation (Singh and Mijakovic, 2022). While not within the scope of this study, the potential cytotoxicity and biocompatibility of AgNPs are important considerations for their therapeutic applications. Future research should address these concerns, including studies on genotoxicity and hemocompatibility, as well as exploring the potential for combination therapy with traditional antibiotics to enhance efficacy and reduce resistance. These nanoparticles’ size, concentration, and stability affect their effectiveness; smaller nanoparticles typically exhibit more activity. To fully utilise AgNPs in the therapeutic and commercial domains, future studies should concentrate on refining synthesis parameters, understanding intricate antibacterial mechanisms, assessing clinical applications, and resolving environmental and regulatory issues (Sastry et al., 1997). Thus, the synthesis of AgNPs using IAA and IBA demonstrates a new and environmentally friendly method and highlights the potential of these nanoparticles as potent antibacterial agents. Further studies may concentrate on refining the conditions of synthesis to enhance the characteristics of nanoparticles and exploring their potential uses in diverse domains like healthcare, farming, and environmental restoration.

## 5 Conclusion

This study establishes a green synthesis route for stable, well-characterised AgNPs using IAA and IBA as reducing and stabilising agents. The synthesised AgNPs exhibited significant antibacterial activity, with *E. coli* showing higher susceptibility than *S. aureus*, due to differences in cell wall structure and silver ion uptake. These findings demonstrate the strong antimicrobial potential of IAA/IBA-mediated AgNPs, supporting their future application in sustainable and effective antibacterial therapies.

## Data Availability

The original contributions presented in the study are included in the article/supplementary material. Further inquiries can be directed to the corresponding author.
