# Correction: Auxin-mediated biosynthesis of silver nanoparticles: comprehensive characterisation and antibacterial activity analysis

**DOI:** 10.3389/fcimb.2025.1763110

**Published:** 2026-01-12

**Authors:** Sahil, Anis Ahmad Chaudhary, Sonia Sorout, Kushi Yadav, Mohamed A. M. Ali, Fehmi Boufahja, Vikram Kumar, SL Kothari, Devendra Jain, Kumar Sambhav Verma

**Affiliations:** 1 Amity Institute of Biotechnology, Amity University Rajasthan, Jaipur, Rajasthan, India; 2 Department of Biology, College of Science, Imam Mohammad Ibn Saud Islamic University (IMSIU), Riyadh, Saudi Arabia; 3 Amity Institute of Biotechnology, Amity University Uttar Pradesh, Noida, Uttar Pradesh, India; 4 Amity Institute of Pharmacy, Amity University Rajasthan, Jaipur, Rajasthan, India; 5 Department of Molecular Biology and Biotechnology, Maharana Pratap University of Agriculture & Technology, Udaipur, Rajasthan, India

**Keywords:** nanoparticles, antimicrobial activity, antibiotic, indole-3-acetic acid, indole-3-butyric acid, green nanotechnology, auxin-mediated synthesis

There was a mistake in the caption of **Figures 3**, **4**, **5** as published. Figure captions error for **Figures 3**, **4**, **5**. The corrected caption of **Figures 3**, **4**, **5** appears below.

**Figure 3:**
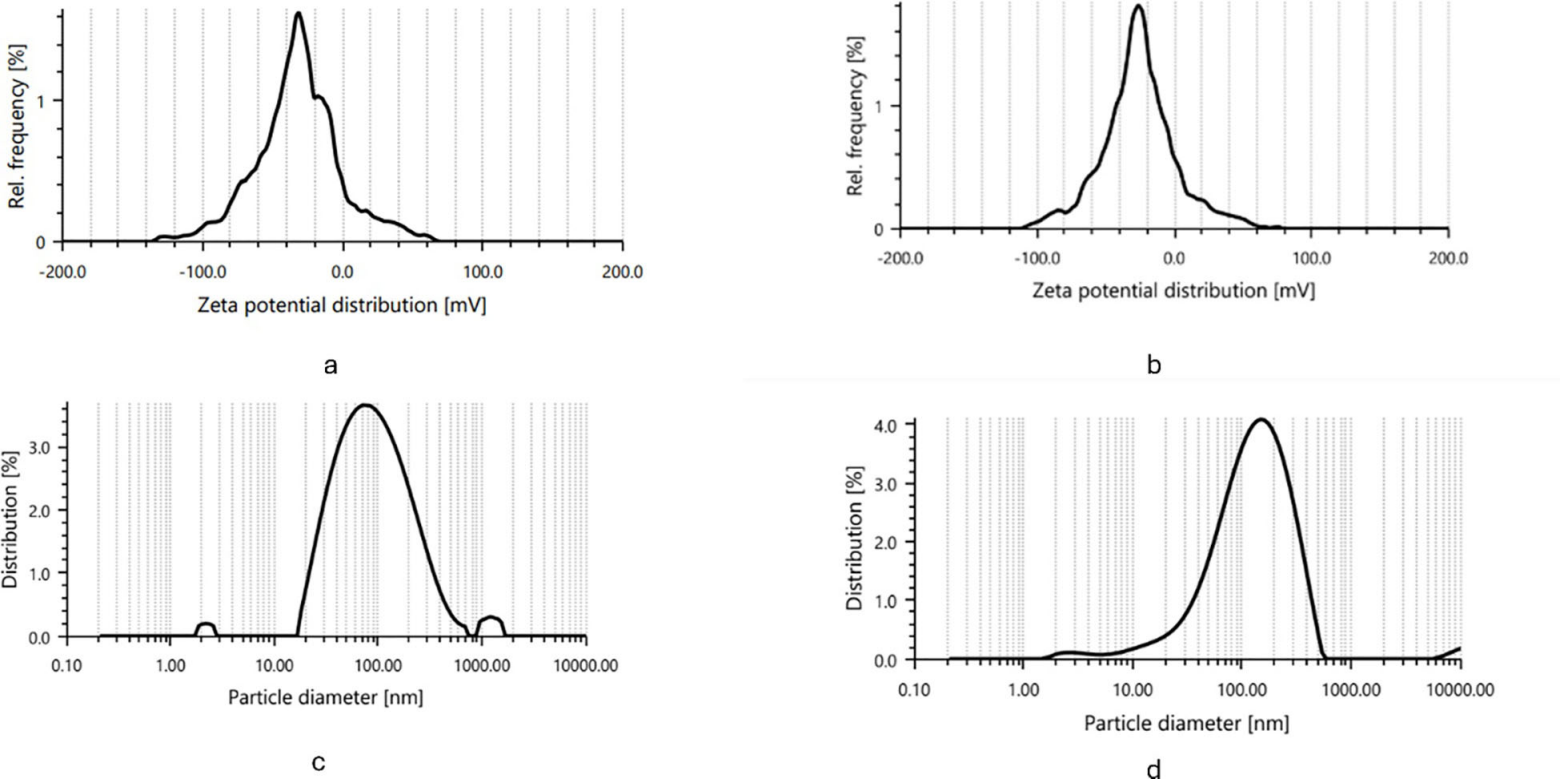
Zeta potential and particle size distribution of synthesized silver nanoparticles. **(a, c)** IAA -AgNPs and **(b, d)** IBA -AgNPs showing stable zeta potential and uniform particle size distribution in the nanometer range.

**Figure 4:**
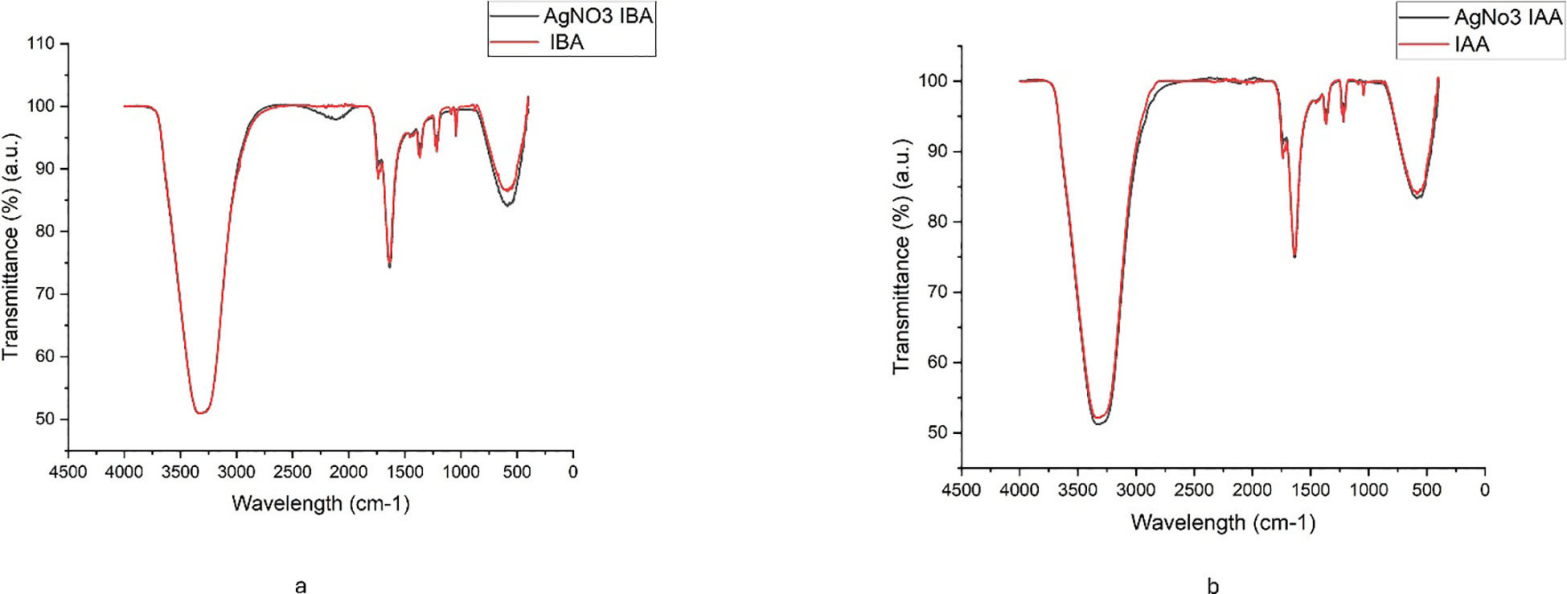
FTIR spectra of **(a)** IBA and AgNO_3_ -IBA, and **(b)** IAA and AgNO_3_ -IAA, showing characteristic functional groups involved in silver nanoparticle synthesis and stabilization.

**Figure 5:**
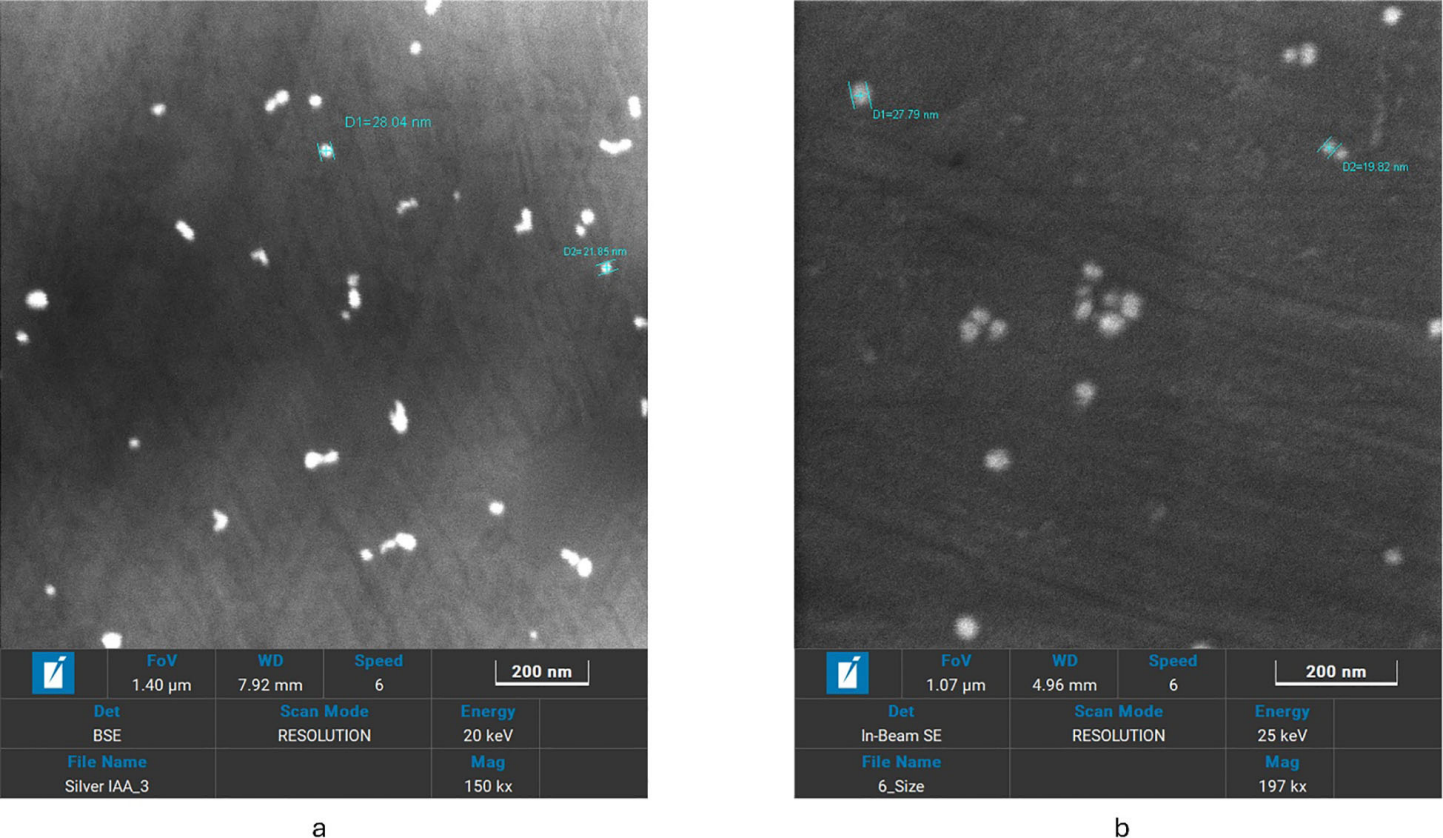
SEM images of **(a)** IAA -AgNPs and **(b)** IBA -AgNPs showing uniformly dispersed nanoparticles.

An incorrect **Funding** statement was provided. The correct **Funding** statement reads:

“This work was supported and funded by the Deanship of Scientific Research at Imam Mohammad Ibn Saud Islamic University (IMSIU) (grant number IMSIU-DDRSP2501).”

An incorrect **Acknowledgements** was provided. The correct **Acknowledgements** reads:

“This work was supported and funded by the Deanship of Scientific Research at Imam Mohammad Ibn Saud Islamic University (IMSIU) (grant number IMSIU-DDRSP2501).

We are also thankful to DST FIST, DST Purse labs of Amity Institute of Biotechnology, Amity University Rajasthan Jaipur for providing the needful research facilities. We want to thank UGC New-Delhi, for providing SRF to Sahil.”

The original version of this article has been updated.

